# Rehydrated Air-Dried Pap Smears as a Cost-Effective Alternative to Conventional Cytology in Low-Resource Settings: A Cytomorphological Comparative Study

**DOI:** 10.7759/cureus.110284

**Published:** 2026-06-05

**Authors:** Priyanka Hembram, Manoj K Paswan, Sunil Kumar Mahto, Prachi Hansdah, Anshu Jamaiyar

**Affiliations:** 1 Pathology, Rajendra Institute of Medical Sciences, Ranchi, IND; 2 Ophthalmology, Rajendra Institute of Medical Sciences, Ranchi, IND

**Keywords:** bethesda reporting system, cervical cytology, cervical lesions, conventional papanicolaou (pap) smear, rehydrated air-dried smears, wet-fixed smear

## Abstract

Background: Cervical cancer is still a significant public health problem, especially in low- and middle-income countries. Rehydration of air-dried smears (RADS) has been developed as an alternative technique to overcome limitations associated with conventional wet fixation. The present study was undertaken to compare rehydrated air-dried Pap smears with conventional wet-fixed smears (WFS) for evaluation of cervical lesions, with emphasis on smear adequacy, cytomorphological features, and diagnostic concordance using the Bethesda system.

Methods: A hospital-based cross-sectional comparative study was conducted on 282 women undergoing cervical cytology. Three smears were prepared from each sample: one conventional WFS and two rehydrated air-dried smears. Smears were stained using the standard Papanicolaou technique and evaluated for adequacy, cellularity, background features, cytolysis, artifacts, nuclear features, cytoplasmic staining, and diagnostic concordance using the Bethesda system.

Results: Rehydrated air-dried smears demonstrated higher adequacy rates than WFS (275 (97.5%) vs 254 (90.1%)). Red blood cell obscuration (13 (4.6%) vs 127 (45.0%)), cytolysis (11 (3.9%) vs 33 (11.7%)), and air-drying artifacts (15 (5.3%) vs 50 (17.7%)) were significantly reduced in rehydrated smears. Nuclear borders (263 (93.3%) vs 245 (86.9%)) and chromatin quality (271 (96.1%) vs 234 (83.0%)) were better preserved in rehydrated smears. Diagnostic concordance between WFS and rehydrated smears was high at 276 (97.9%), with a Cohen's kappa value of 0.975 indicating almost perfect agreement.

Conclusion: Rehydrated air-dried Pap smears performed similarly to conventional WFS in the diagnosis of cervical lesions. The technique showed better adequacy, cleaner background, satisfactory nuclear and cytoplasmic detail, and high diagnostic concordance. Rehydrated air-dried smears could be a practical and efficacious replacement for standard wet fixation, particularly in resource-limited settings.

## Introduction

Cervical cancer is still a significant public health problem, especially in low- and middle-income countries, accounting for one of the highest cancer morbidity and mortality in women [1,2]. Persistent infection with high-risk human papillomavirus (HPV) is the main cause of cervical carcinogenesis, which may last for several years and culminate in the development of invasive carcinoma, after the passage of several identifiable pre-invasive stages: low-grade squamous intraepithelial lesion (LSIL) and high-grade squamous intraepithelial lesion (HSIL) [3,4]. This extended period of pre-invasive disease is an important window for early detection via cytological screening, especially as a large proportion of cervical cancers occurs in developing countries, where access to effective screening and early diagnostic services is limited [5]. The Pap smear plays a crucial role in decreasing the burden of cervical cancer through well-organised screening programmes [6].

Conventional Pap smear preparation requires immediate wet fixation in 95% ethanol to optimize the nuclear and cytoplasmic detail [7-9]. While this is effective, in many high-volume outpatient departments and peripheral health care facilities, logistical issues and the lack of the required fixative can make immediate wet fixation impractical, producing artifacts such as nuclear distortion, chromatin smudging, and changes to the cytoplasm that can obscure the epithelial cell and diminish diagnostic value [10]. Blood and inflammatory exudates can also hide the epithelial cells and decrease the yield of the Pap smear [11]. To overcome these limitations, rehydration of air-dried smears (RADS) has been developed as an alternative technique. In addition, RADS seems beneficial in resource-poor environments and outreach screening programs that require time for immediate fixation of smears.

Despite encouraging findings, existing literature shows variability in rehydration protocols, assessment parameters, and reported diagnostic concordance. While several studies report comparable detection of epithelial abnormalities, others note minor limitations in subtle high-grade lesions. In view of these considerations, the present study was undertaken to compare rehydrated air-dried Pap smears with conventional wet-fixed smears (WFS) for evaluation of cervical lesions, with emphasis on smear adequacy, cytomorphological features, and diagnostic concordance using the Bethesda system.

## Materials and methods

The required minimum sample size for our comparison study was 256, using the expected prevalence of 1.7%, an absolute precision of 5%, and a confidence interval of 95% [11]. This is a standard sample size estimation used in epidemiological research and is sufficient to achieve adequate power to detect significant differences in diagnostic parameters. Females aged 25-75 years from low socio-economic status attending the Gynecology Department at Rajendra Institute of Medical Sciences (RIMS), Ranchi, India, with symptoms such as abdominal pain, prolonged bleeding per vaginum, itching, white vaginal discharge, or prolapse were included in the study. Pregnant or menstruating women, post-hysterectomy patients, and those unwilling to participate were excluded. Consecutive sampling was used, and a final sample population of 282 was included to strengthen the analysis.

Cervical samples were collected under aseptic precautions using an Ayre's spatula and/or endocervical brush to ensure that the transformation zone was adequately sampled in each participant. On clean glass slides, three smears were prepared. One smear was immediately fixed in 95% ethanol and processed as a traditional WFS. The other smears were left to air dry at room temperature (about 30 minutes). These air-dried smears were then rehydrated in 0.9% normal saline for 30 seconds to 2 minutes and immediately fixed in 95% ethanol.

The standard Papanicolaou staining technique was used to stain all smears. Slide interpretation was performed in a blinded manner, with the pathologist evaluating RADS and another evaluating routine WFS independently without knowledge of the corresponding smear results. Microscopic analysis was conducted under light microscopy, and smears were evaluated based on adequacy, cellularity, background features (presence of red blood cells and inflammatory cells), cytolysis, air-drying artifacts, nuclear features, and quality of cytoplasmic staining. Cytological interpretation was done according to the Bethesda System of Reporting Cervical Cytology (2014) [12].** **Paired comparison of WFS and RADS was done for each case, and diagnostic concordance between the two methods was documented. The data were entered into an Excel Sheet (Microsoft® Corp., Redmond, WA) and analyzed using Statistical Product and Service Solutions (SPSS, version 27; IBM SPSS Statistics for Windows, Armonk, NY). Categorical data were expressed as frequencies and percentages. The McNemar test was used for statistical comparison, and Cohen's kappa was used for assessing diagnostic agreement. A p-value of < 0.05 was considered statistically significant.

The study was approved by the Institutional Ethics Committee (IEC No. 89 dated 01/02/2025). All patient data were anonymized for analysis, and patient confidentiality was respected.

## Results

**Figure 1:**
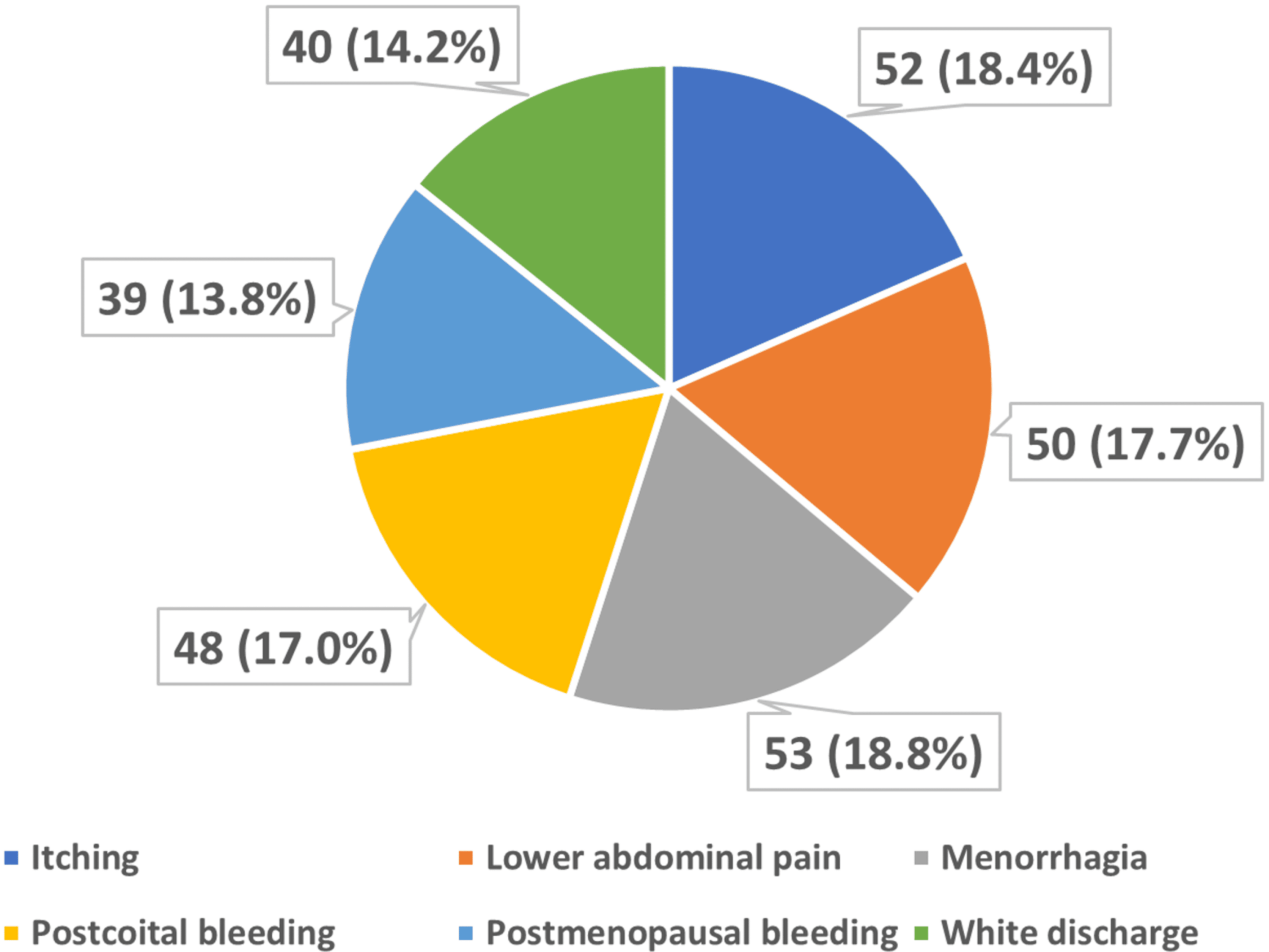
Distribution of complaints among study participants (n = 282)

A total of 282 women undergoing cervical cytology were included in the study. The age of participants ranged from 25 to 75 years, with a mean age of 49.96 ± 13.99 years. Women aged 25-50 years constituted 150 (53.2%) of the study population, while 132 (46.8%) were aged 50-75 years. Menorrhagia (53, 18.8%), itching (52, 18.4%), and lower abdominal pain (50, 17.7%) were the most common presenting complaints (Figure 1).

RADS demonstrated higher adequacy rates than WFS (275 (97.5%) vs 254 (90.1%)), with significantly fewer unsatisfactory smears in the RAD group (7 (2.5%) vs 28 (9.9%)). High cellularity was observed more frequently in RADS (143, 50.7%) than in WFS (87, 30.9%), whereas low cellularity was more common in WFS preparations (109, 38.7%). Endocervical cell clusters showing the smear adequacy are presented in Appendix 6. The difference in smear adequacy was statistically significant on McNemar analysis (p < 0.001), while cellularity did not show a statistically significant difference (Table 1).

**Table 1:** Comparison of smear adequacy and cellularity between techniques *McNemar χ², ^#^Chi-square test

Parameter	Category	Wet-Fixed, n (%)	Rehydrated, n (%)	χ² value (df)	p-value
Smear adequacy	Adequate	254 (90.1)	275 (97.5)	12.12^*^ (1)	<0.001
	Inadequate	28 (9.9)	7 (2.5)		
Cellularity	High	87 (30.9)	143 (50.7)	0.315^#^ (2)	0.854
	Intermediate	86 (30.5)	139 (49.3)		
	Low	109 (38.7)	-		

Background characteristics demonstrated significantly improved smear clarity with the rehydration technique (smear showing inflammatory background and reactive cellular changes in RADS and WFS are shown in Appendices 3 and 4, and visualization of background flora and lactobacilli in WFS and RADS is shown in Appendices 7 and 8. Red blood cell (RBC) obscuration was observed in 127 (45.0%) of WFS compared with 13 (4.6%) of RADS. Cytolysis was lower in RADS than in WFS (11 (3.9%) vs 33 (11.7%)), and air-drying artifacts were also less frequent in RADS (15 (5.3%) vs 50 (17.7%)) (Table 2). An example of an air-drying artifact observed during smear evaluation is shown in Appendix 5.

**Table 2:** Comparison of background characteristics and artifacts *McNemar χ²

Parameter	Category	Wet-Fixed, n (%)	Rehydrated, n (%)	McNemar χ² (df)	p-value
RBC background	Present	127 (45.0)	13 (4.6)	104.03 (1)	<0.001
	Absent	155 (55.0)	269 (95.4)		
Cytolysis	Present	33 (11.7)	11 (3.9)	10.08 (1)	0.001
	Absent	249 (88.3)	271 (96.1)		
Artifacts	Present	50 (17.7)	15 (5.3)	18.85 (1)	<0.001
	Absent	232 (82.3)	267 (94.7)		

Cytomorphological assessment revealed that nuclear and chromatin details were significantly better in RADS. High-grade squamous intraepithelial lesion (HSIL) morphology between WF and RAD smears is shown in Appendices 9 and 10. Observation of distinct nuclear borders in 263 (93.3%) of RADS versus 245 (86.9%) of WFS, as well as crisp chromatin in 271 (96.1%) and 234 (83.0%), respectively. The results showed satisfactory cytoplasmic staining in both techniques (RADS of 275 (97.5%) and conventional smears of 268 (95.0%)), with better staining rates for RAD (Table 3).

**Table 3:** Cytomorphological features comparison between WFS and RADS *McNemar χ² RADS: Rehydration of air-dried smears; WFS: Wet-fixed smears

Parameter	Category	Wet-Fixed, n (%)	Rehydrated, n (%)	McNemar χ² (df)	p-value
Nuclear border	Distinct	245 (86.9)	263 (93.3)	7.31 (1)	0.007
	Indistinct	37 (13.1)	19 (6.7)		
Chromatin quality	Crisp	234 (83.0)	271 (96.1)	30.02 (1)	<0.001
	Hazy	48 (17.0)	11 (3.9)		
Cytoplasmic staining	Adequate	268 (95.0)	275 (97.5)	4.57 (1)	0.033
	Inadequate	14 (5.0)	7 (2.5)		

**Figure 2:**
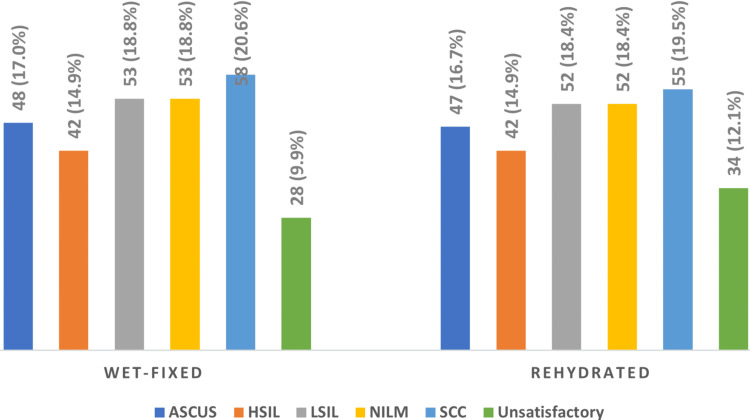
Distribution of cytological diagnosis (Bethesda system)

According to Bethesda System 2014 [12], squamous cell carcinoma (58 (20.6%) vs 55 (19.5%)), low-grade squamous intraepithelial lesion (53 (18.8%) vs 52 (18.4%)), and negative for intraepithelial lesion or malignancy (53 (18.8%) vs 52 (18.4%)) were the predominant diagnostic categories in WFS and RADS, respectively (Figure 2).

Diagnostic concordance between WFS and RADS was high at 276 (97.9%), with discordance observed in only six (2.1%) cases. The Cohen's kappa value was 0.975, indicating almost perfect agreement between the two techniques. This good concordance supports the reliability and concordance of rehydrated air-dried smears as another option to traditional wet-fixed preparations in cervical cytology.

## Discussion

A total of 282 patients underwent systematic comparison between RAD Pap smears and routine WFS, revealing a high agreement rate of 97.9%, with a Cohen's kappa value of 0.975 indicating almost perfect agreement between the two techniques. Similar concordance rates were reported by Jaiwong et al. and supported by previous studies demonstrating high concordance between RADS and WFS for the detection of epithelial lesions [10]. Among RADS, smear adequacy was significantly higher (97.5%) than WFS (90.1%), with fewer unsatisfactory smears in the RADS group. Gupta et al. also demonstrated improved adequacy, possibly due to better preservation of cellular integrity, while Sivaraman et al. reported reduced air-drying artifacts in rehydrated smears [9,13]. Similar improvements in smear adequacy and cellular preservation were also reported by Kapse et al. and Kamble et al. [14,15]. Our study showed a statistically significant increase in satisfactory smears, suggesting that the rehydration technique may reduce repeat sampling and improve smear quality.

One of the important findings of this study was the markedly improved background clarity in RADS. RBC obscuration decreased significantly from 45.0% in WFS to 4.6% in RADS, indicating effective hemolysis during saline rehydration. Similar findings were reported by Kapse et al. and Kamble et al., who observed improved background clarity and better visualization of epithelial cells following rehydration [14,15]. Cytolysis and air-drying artifacts were also significantly reduced in RADS. Similar findings were observed by Narayanan et al., who demonstrated decreased artifact formation in rehydrated smears [11]. Rupinder et al. further highlighted that controlled rehydration restores cellular morphology and reduces alterations induced by air-drying [8]. Danquah et al. also observed improved cytomorphological preservation and reduction of preparation-related artifacts with alternative fixation methods in cervical cytology [16]. These findings suggest that smear quality is improved by reducing obscuring background elements and preparation-related artifacts.

Our cytomorphological evaluation revealed significant improvement in nuclear and chromatin detail in RADS, with distinct nuclear borders (93.3% in RAD vs 86.9% in WF) and crisp chromatin (96.1% in RAD vs 83.0% in WF). Satisfactory cytoplasmic staining was also slightly higher in RADS. Gupta et al. and Jaiwong et al. similarly reported improved nuclear clarity and chromatin detail in rehydrated smears [9,10]. However, Gupta et al. reported occasional chromatin haziness in borderline lesions, which may reflect variability in fixation and rehydration protocols [9]. The significant cytomorphological improvement observed in our study further supports the applicability of the rehydration technique in cervical cytology. Similar observations regarding nuclear preservation and smear quality were documented by Bamanikar et al. in their comparative evaluation of cervical smear quality parameters [17].

The distribution of diagnostic categories based on the Bethesda classification in both approaches was similar. Consistent with the findings reported above, RADS were consistently categorized with a highly similar proportion of squamous cell carcinoma, LSIL, and NILM to the respective WFS, demonstrating that rehydration does not affect cytological categorization. The similar findings were recorded in the study conducted by Mirzaie et al. and Shidham et al., who mentioned that cytomorphology after rehydration smears remains adequate for Bethesda-based reporting [18,19]. Overall concordance was high, with only 2.1% of cases demonstrating discordance. Prior studies have also reported similar discordance rates of 2%-5%, indicating that this variability is primarily due to cytological interpretation rather than pitfalls of the rehydration technique. These positive findings of the study have implications for cervical cancer screening, particularly in low-resource settings where wet fixation cannot always be performed immediately. Overall, the rehydration method displayed significantly better smear adequacy, much improved background clarity (decreased artifact), and high diagnostic concordance with no compromise of cytological interpretation. These benefits may lead to improved smear quality, fewer repeat biopsies, and more effective cervical cancer screening programs in both peripheral and high-volume healthcare settings.

The strengths of this study include the paired design, adequate sample size, and standardized evaluation using the Bethesda system. Multiple cytological parameters, including adequacy, cellularity, background characteristics, and cytomorphological features, were systematically assessed. However, limitations include the single-center design, evaluation by a single observer, and lack of consistent histopathological correlation, which may limit generalizability and definitive validation of cytological findings.

## Conclusions

Our study demonstrated that RADS performed similarly to conventional WFS in the diagnosis of cervical lesions. RADS showed much better adequacy, decreased obscuring background caused by significant lysis of RBCs, and overall observed satisfactory nuclear and cytoplasmic details. Our study showed almost perfect agreement between RADS and the conventional WFS, confirming that rehydrated smears performed using the Bethesda system are reliable cytological interpretation methods for clinical use. Our study concludes that the RADS could be a practical and efficacious replacement for the standard wet fixation, particularly when immediate fixation is imperative.

## Figures and Tables

**Figure 3:**
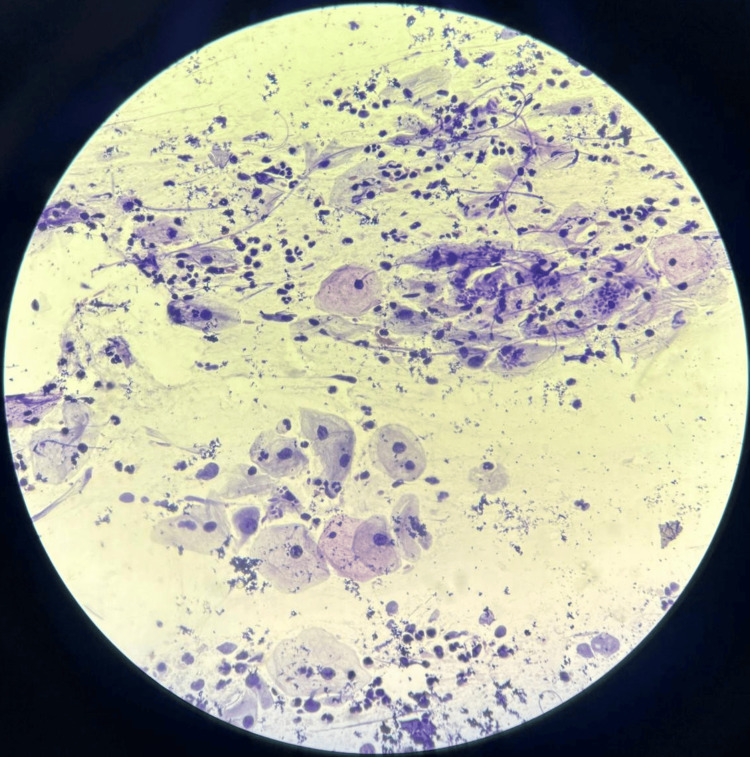
Appendix 3: Inflammatory smear with reactive changes, ADRS (Papanicolaou stain, 400x) ADRS: Atypical degenerative reactive squamous

**Figure 4:**
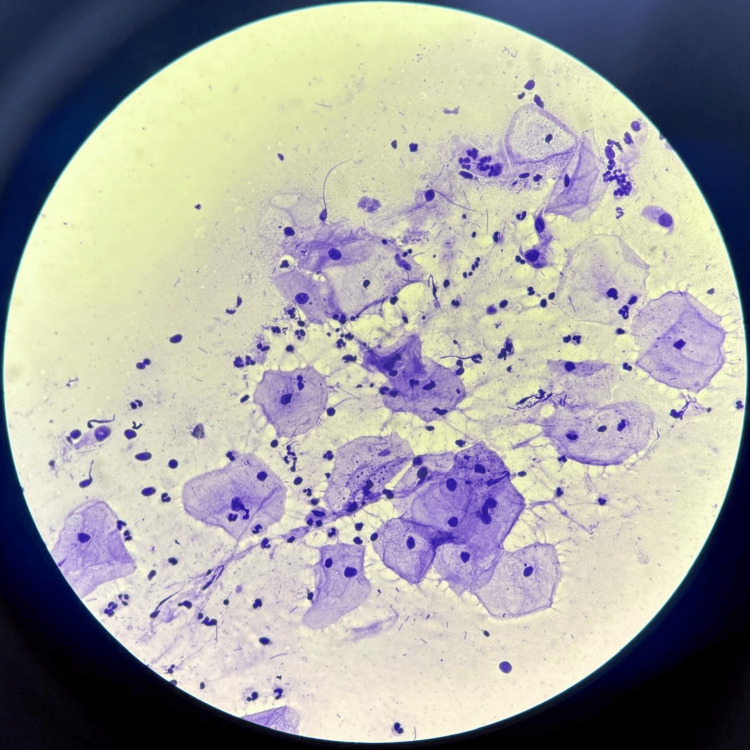
Appendix 4: Inflammatory smear, WFS (Papanicolaou stain, 400x) WFS: Wet-fixed smears

**Figure 5:**
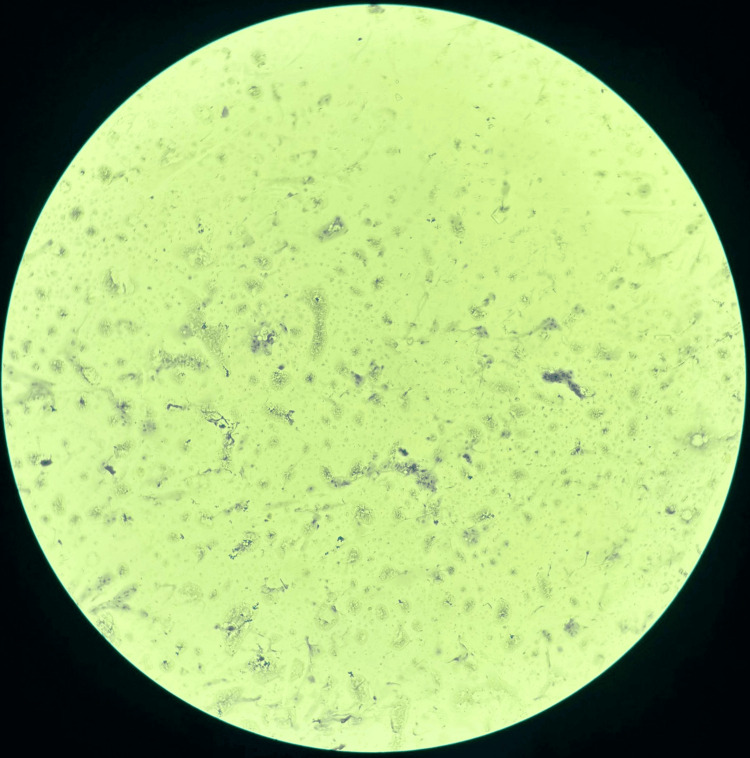
Appendix 5: Presence of air-drying artifact in ARDS (100x) ARDS: Atypical degenerative reactive squamous

**Figure 6:**
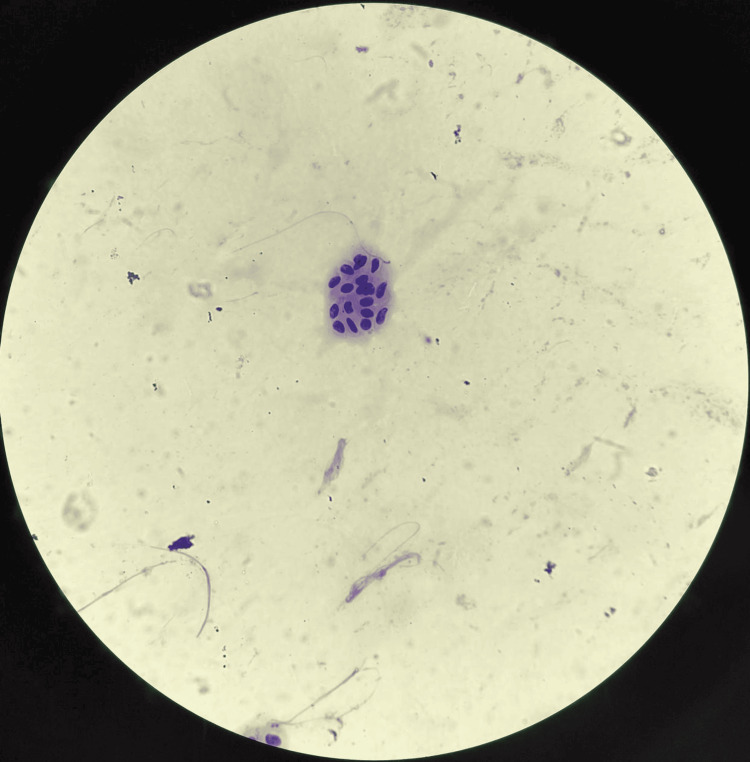
Appendix 6: Presence of endocervical cluster (WFS) (400x) WFS: Wet-fixed smears

**Figure 7:**
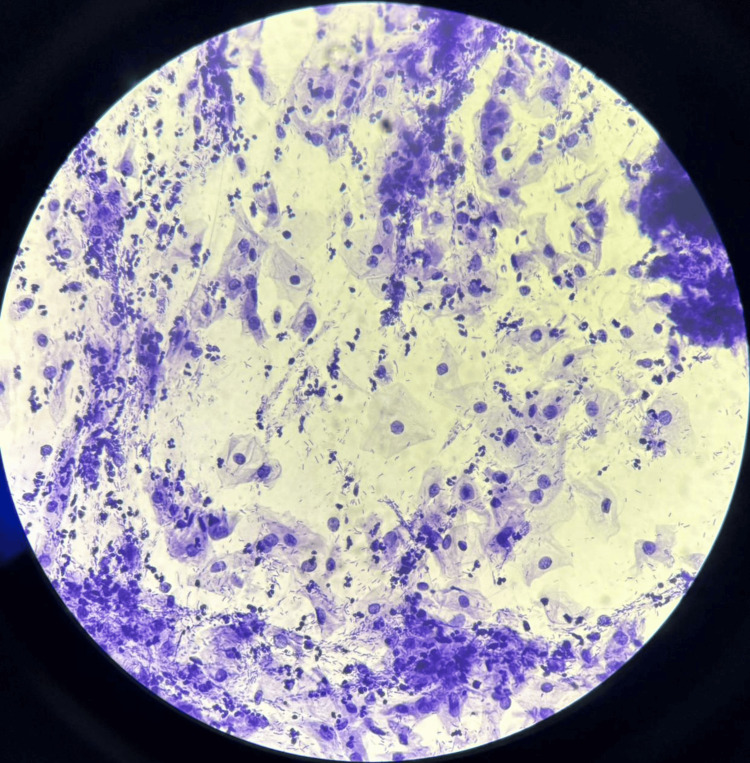
Appendix 7: Presence of lactobacilli, WFS (Papanicolaou stain, 400x) WFS: Wet-fixed smears

**Figure 8:**
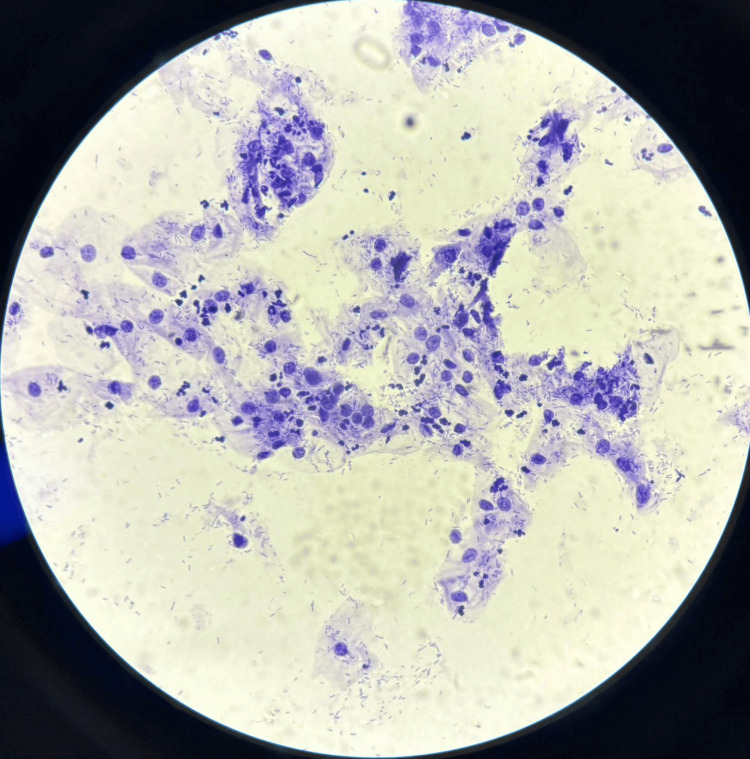
Appendix 8: Presence of lactobacilli, ADRS (Papanicolaou stain, 400x) ADRS: Atypical degenerative reactive squamous

**Figure 9:**
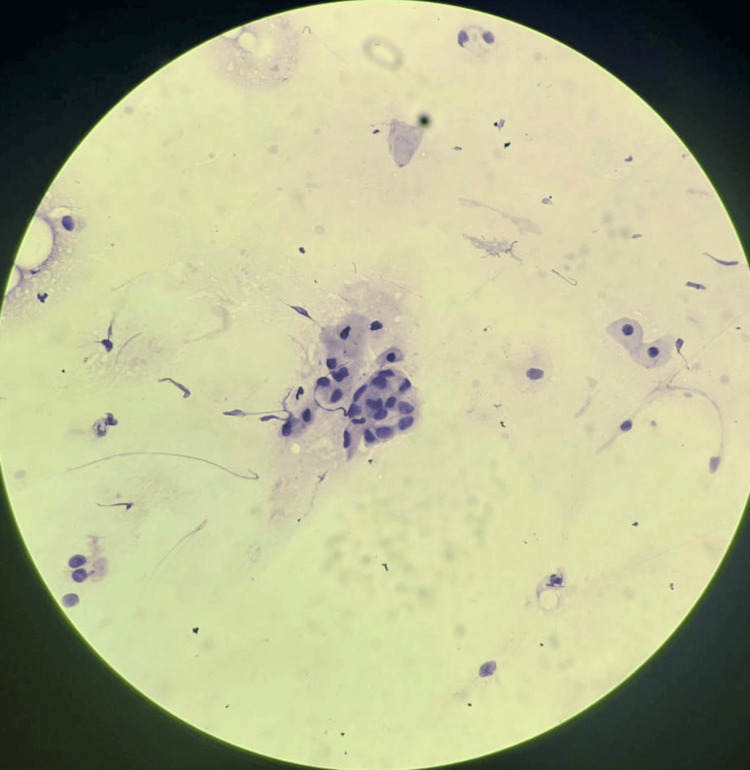
Appendix 9: High-grade squamous intraepithelial lesion, WFS (Papanicolaou stain, 400x) WFS: Wet-fixed smears

**Figure 10:**
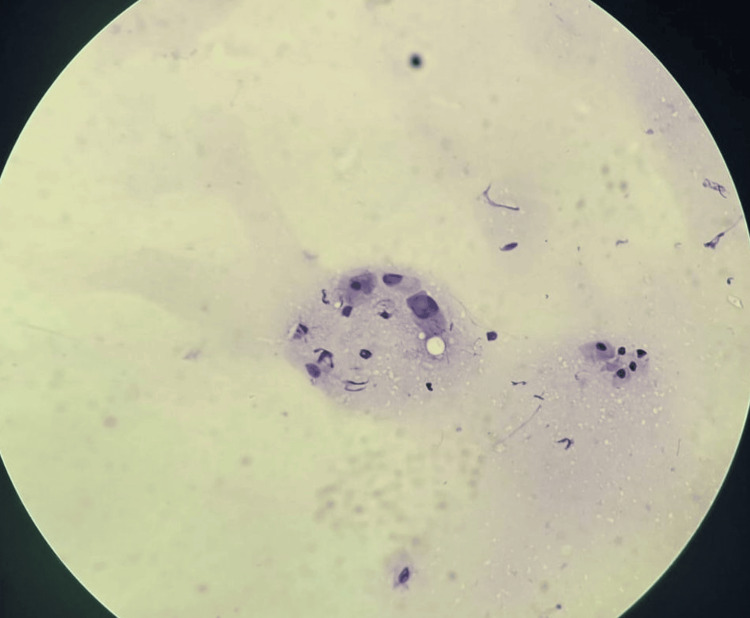
Appendix 10: High-grade squamous intraepithelial lesion, ADRS (Papanicolaou stain, 400x) ADRS: Atypical degenerative reactive squamous

## References

[1] Ferlay J, Soerjomataram I, Dikshit R (2015). Cancer incidence and mortality worldwide: sources, methods and major patterns in GLOBOCAN 2012. Int J Cancer.

[2] Bobdey S, Sathwara J, Jain A, Balasubramaniam G (2016). Burden of cervical cancer and role of screening in India. Indian J Med Paediatr Oncol.

[3] Schiffman M, Castle PE, Jeronimo J, Rodriguez AC, Wacholder S (2007). Human papillomavirus and cervical cancer. Lancet.

[4] Crosbie EJ, Einstein MH, Franceschi S, Kitchener HC (2013). Human papillomavirus and cervical cancer. Lancet.

[5] Sreedevi A, Javed R, Dinesh A (2015). Epidemiology of cervical cancer with special focus on India. Int J Womens Health.

[6] Rayner M, Welp A, Stoler MH, Cantrell LA (2023). Cervical cancer screening recommendations: now and for the future. Healthcare (Basel).

[7] Bukhari MH, Saba K, Qamar S, Majeed MM, Niazi S, Naeem S (2012). Clinicopathological importance of Papanicolaou smears for the diagnosis of premalignant and malignant lesions of the cervix. J Cytol.

[8] Rupinder K, Shubra W, Kanwal M (2013). Rehydration of air-dried smears versus wet fixation: a cross-sectional study. Acta Cytol.

[9] Gupta S, Sodhani P, Chachra KL (2003). Rehydration of air-dried cervical smears: a feasible alternative to conventional wet fixation. Obstet Gynecol.

[10] Jaiwong K, Nimmanhaeminda K, Siriaree S, Khunamornpong S (2006). The cytomorphologic comparison between rehydrated air-dried and conventional wet-fixed pap smears. J Med Assoc Thai.

[11] Narayanan ON, Bai A (2019). Comparison of different methods of fixation in Papanicolaou staining of cervical smears: wet-fixation and rehydration of air dried smears. Trop J Path Micro.

[12] Nayar R, Wilbur DC (2015). The Pap test and Bethesda 2014. Cancer Cytopathol.

[13] Shidham VB, Kampalath B, England J (2001). Routine air drying of all smears prepared during fine needle aspiration and intraoperative cytology studies. An opportunity to practice a unified protocol offering the flexibility of choosing a variety of staining methods. Acta Cytol.

[14] Kapse SS, Arakeri SU, Yerranguntla DP (2018). Rehydration of air-dried smears with normal saline: an alternative for conventional wet fixation method in cervical cytological study. J Cytol.

[15] Kamble SA, Dombale VD, Shah F (2022). Merits and pitfalls of normal saline rehydrated air-dried cervical smears over conventional wet-fixed PAP smears: a comparative study. Indian J Pathol Microbiol.

[16] Danquah KO, Adankwah E, Dadzie HE, Gyamfi D, Adjei EA, Ossei Sampene PP, Morhe E (2023). Prolonged duration of air-dry fixation of cervical smears produces superior cytomorphological staining quality over conventional wet-fixed smears. Acta Cytol.

[17] Bamanikar SA, Baravkar DS, Chandanwale SS (2018). Study of cervical Pap smears in a tertiary hospital and comparison of smear quality parameters. Med J Dr DY Patil Vidyapeeth.

[18] Sivaraman G, Iyengar KR (2002). Rehydrated air-dried Pap smears as an alternative to wet-fixed smears. Acta Cytol.

[19] Zare-Mirzaie A, Khalili-Alam K, Abolhasani M (2007). Rehydration of air-dried cervical smears: an alternative to routine wet fixation. Acta Med Iran.

